# From storytelling to scripting: eight principles for deconstructing crime events

**DOI:** 10.1186/s40163-026-00286-w

**Published:** 2026-06-15

**Authors:** Hervé Borrion, Paul Ekblom, Alexandre Bish, Congyu Fan, Juliana Gómez-Quintero, Peng Chen, Sonia Toubaline, Alina Ristea, Manny Niri

**Affiliations:** 1 UCL Department of Security and Crime Science, University College London, 35 Tavistock Square, London, WC1H 9EZ UK; 2 Design against Crime Research Lab, Central Saint Martins, University of the Arts, London, UK; 3 School for Informatics Cyber Security, People’s Public Security University of China, Beijing, People’s Republic of China; 4 LAMSADE, Université Paris-Dauphine, PSL University, CNRS, 75016 Paris, France; 5 School of Engineering, Computing and Mathematics, Oxford Brookes University, Oxford, UK

## Abstract

This paper aims to improve crime script analysis, with a particular focus on breaking script stages into smaller, more precise steps. After outlining existing scripting methods, we then introduce eight principles designed to guide both the creation of crime scripts and the evaluation of scripts produced by others. Grounded in the concepts of state and activity, these principles address the following themes: Constitution, Chronology, Composition, Cardinality, Categorisation, Completeness, Clarity, and Consistency. Finally, we discuss the limits of template based approaches, including those built around the universal script. We call for the development of a more flexible scripting method, one that retains the chronological and functional cues of the universal script, but can be applied at any level of abstraction and resolution. Within this context, we believe the above principles can play another important role in its development.

**Supplementary Information:**

The online version contains supplementary material available at 10.1186/s40163-026-00286-w.

## Introduction

Understanding how crime occurs is key to effective crime prevention and detection (Haelterman, 2016). Ekblom (2014) for example, suggests ‘thinking thief’: placing oneself in the shoes of the offender. Also referred to as adversarial thinking, this approach has known considerable success in related fields, including cybersecurity (Schneider, 2013) and fraud prevention (Iyer & Samociuk, 2016). Numerous techniques have been proposed to support this form of thinking, many of which overlap conceptually. These include attack graphs, attack trees, misuse cases, abuse cases, attack-defence trees and crime scripts. By describing the processes offenders follow to commit crimes, these techniques can greatly support practitioners. Crime script analysis (CSA), a systematic analytical approach for generating, organising, and structuring knowledge about the procedural steps, decision points, and enabling conditions involved in the commission of a crime, is particularly useful to identify a broad range of ‘pinch points’ at which tailored crime reduction interventions may be introduced (Cornish 1994b; Dehghanniri and Borrion 2021).

Three decades after Cornish’s (1994a, Cornish 1994b) seminal papers, CSA is progressively reaching mainstream status in scientific publications and government reports. Users are believed to include not only university researchers but also criminal justice system practitioners. The UK College of Policing (2022), for instance, produced a video explaining how crime scripts should be developed to reveal the procedural aspects of crime and support crime prevention and disruption efforts by the police. The United Nations also recommends using crime scripts to address crimes such as procurement fraud or wildlife crime (Darioli et al., 2021; UNODC, 2024).

CSA has been applied to a diverse range of crime problems including drug production, sexual offending, cyber-enabled fraud, organised crime markets, wildlife crime and irregular migration (e.g., Beauregard et al., 2007; Bish et al., 2024; Chiu et al., 2011; Gómez-Quintero et al., 2026; Leclerc et al., 2011; Loggen & Leukfeldt, 2022; Tsai et al., 2024). In the academic literature, scripts are typically developed from sources such as interviews with offenders, victims, and security practitioners (e.g., Beauregard et al., 2007; Pires & Solans, 2023; Skidmore, 2021; Smith, 2017), investigative records (e.g., Carney et al., 2023; De Korte & Kleemans, 2022), court cases (e.g., Chiu et al., 2011; O’Hara et al., 2020), or other forms of open-source intelligence (e.g., Borrion et al., 2017; Chainey & Alonso Berbotto, 2021; Gondhali et al., 2024; Stickle et al., 2022).

Despite the growing body of empirical applications, relatively limited attention has been paid to the methodological aspects of script construction. Cornish (1994a, b)’s publications contain the majority of the today’s knowledge about the make-up of crime scripts (e.g., structure, abstraction levels, types of elements, etc.). For their elaboration, he initially recommends using a template known as the universal script and composed of generalised stages called scenes. There appears to be some confusion about this template. For instance, Riungu et al. (2025, p. 2) indicate that the universal script includes ‘seven successive stages’ while O’Hara et al. (2020, p. 4) report ‘eight script scenes/functions’. As described later, the template includes nine scenes, although it was suggested to increase it to 10. Lemieux and Bruschi (2019), for instance, added a final scene called Aftermath.

Considering Cornish’s (1994a, Cornish 1994b) framework too academic and difficult to operationalise, Tompson and Chainey (2011) streamlined it to make it more accessible for practitioners. The revised template and method was further developed by Chainey and Alonso Berbotto (2021), specifically for open-source intelligence (i.e. publicly available secondary data). The main change, discarding the universal script, proved a popular choice, and many scripts were subsequently created without following the functional template. A search in this journal (keyword: ‘crime script’) returned 19 articles published since its creation (12/2012-05/2026). Among the 11 studies that involved actual production of one or more scripts, the five most recent ones are all based on the classification method proposed by Tompson and Chainey (2011), against four that appear to have been inspired by Cornish’s (1994a, Cornish 1994b) framework and the universal script.

Not all scripts are created using a template-based approach. We found considerable variation among those that don’t. Pointing out that there were ‘no commonly accepted rules on creating scripts’, Brayley et al. (2011) proposed a structured approach to create their script of internal child sex trafficking. Others appear to have adopted a more intuitive approach for extracting information from cases and organising it into a script (e.g., Meyer, 2013).

Although more complex, the universal script offers a useful starting point for breaking down crime events, as does the methodological guidance for information extraction offered by Tompson and Chainey (2011) or the revised version by Chainey and Alonso Berbotto (2021). However, none of them provides a fully systematic method for creating scripts. For example, they offer guidance to identify and classify relevant information, but they do not explain how scenes (or scene classifications) should be logically decomposed into smaller steps, or how steps should be aggregated together into stages.

Regardless of the specific approach used to model the procedural aspects of crime commission, following a procedural template or by extracting a plan from the data themselves, using a structured method or more intuitively, it can be hypothesised that good scripting practice is underpinned by certain principles. Making these principles explicit would be useful to assess scripting methods and inform the development of new ones. In this paper, we propose eight principles for the elaboration of crime scripts. Purposely focused on structural concerns about the decomposition and aggregation of action-units, these principles concern the following aspects: Constitution, Chronology, Composition, Cardinality, Categorisation, Completeness, Clarity, and Consistency. The principles are theoretically justified using constructive counterexamples. We draw on abstract models to illustrate them and discuss the possible consequences of ignoring them. The paper concludes with a discussion about the need to improve existing CSA methods and the ways in which these principles could be validated.

## Crime script analysis

### Crime scripts

Cornish (1994a, b) introduced crime scripts as cognitive tools for organising knowledge about crime and revealing both the ‘procedural aspects’ and ‘procedural requirements’ of crime events. They are described as a special type of schema, known as an event schema, used by offenders to organise knowledge that pertains to understanding and enacting crime-related behavioural processes or routines. As an example, Table 1 represents a burglary script (Cornish 1994a).

**Table 1:** A simple crime script: Burglary (Cornish 1994a)

Acts	Scenes/functions		Activities	
1.	1.1	Preparation	1.1.1	Select a setting
			1.1.2	Assume appropriate role for setting
	1.2	Entry	1.2.1	Enter setting
	1.3	Precondition	1.3.1	Drive around neighbourhood
	1.4	Instrumental precondition	1.4.1	Select target dwelling(s)
	1.5	Exit	1.5.1	Leave setting
2.	2.1	Preparation	2.1.1	Target drugs or alcohol
			2.1.2	Assume appropriate role for setting
	2.2	Entry	2.2.1	Enter the setting
	2.3	Precondition	2.3.1	Wait in vicinity of target dwelling
	2.4	Instrumental precondition	2.4.1	Assess cues relating to surveillability
			2.4.2	Assess cues relating to occupancy
			2.4.3	Assess cues relating to accessibility.
	2.5	Instrumental initiation	2.5.1	Approach dwelling
	2.6	Instrumental actualisation	2.6.1	Break into dwelling
	2.7	Doing	2.7.1	Select and collect up goods
	2.8	Post condition	2.8.1	Leave dwelling with goods
	2.9	Exit	2.9.1	Leave setting

Various key characteristics of crime scripts can be highlighted that are relevant to this paper: procedural decomposition, granularity, descriptive specificity, level of analysis, cast, perspective and type:

*Procedural decomposition* is the primary feature of CSA. Adopting a script-theoretic approach to crime analysis means considering crime not as an instantaneous event but as a process with events happening before and after the main criminal action. A burglary, for instance, involves not only taking the goods, but also other actions including breaking into the dwelling, loading up the stolen goods into a van, and many others.

*Granularity* refers to how finely the sequence of actions is broken down. A step such as ‘break into dwelling’ can be treated as one activity at a coarse resolution or divided into several shorter, sequential actions (e.g., open the bag, grab the brick, look around, look at the window, raise the arm holding the brick, throw the brick in the direction of the window, etc.) at a finer one, while still referring to the same underlying behaviour. Adjusting temporal resolution allows analysts to zoom in or out to match the needs of situational crime prevention, investigative reconstruction, or behavioural analysis.

*Descriptive specificity* (richness) concerns the amount of detail attached to each step of a crime script. A step such as ‘Leave dwelling with goods’ can be enriched by adding information about the action itself (e.g., trajectory and speed of the movement). Whereas granularity alters how many steps the script contains, descriptive specificity alters how much information is embedded within a step. Both dimensions increase the informational content of a script, but they do so in fundamentally different ways.

*Level of analysis* is a topic closely related to the previous points. Described by Cornish (1994a) as the most concrete and crime-specific level, the script track can be generated from one individual crime event or multiple events sharing very strong similarities. Eck and Clarke (2019), in a recapitulation of the rationale for situational crime prevention, state that it has always focused on highly specific categories of crime or disorder, and that understanding exactly how crimes are committed is essential. Families of tracks sharing the same features can be generalised as a script, families of scripts as a protoscript, and families of protoscripts as a metascript. At the highest level of abstraction is the universal script proposed by Leddo and Abelson (1986, p. 118), a sequence of scenes ‘arranged into a sequential order which further the overall action, offer standardised guidelines for constructing scripts at the track level’ (Cornish 1994b, p. 160).

*Cast* refers to the actors, usually individuals, but potentially machines, that take decisions and/or perform actions are directly related to the crime event. Actors can assume one or more roles (e.g., offender, target, guardian, handlers, managers and crime promoters) in the script, not always intentionally (Cohen & Felson, 1979; Leclerc, 2014; Sampson et al., 2010). A teenager who is shot dead while protecting his accomplice during a burglary is an offender, a guardian and a target all at the same time. Individuals can also have multiple civil roles (e.g., his mother can be a homeowner, an accountant and a member of the local church).

*Perspective* refers to the idea that crime commission exists both in the material world and in people’s minds. During a burglary, an offender will be able to rely upon mental models (a.k.a. event schemas) that help them know what to do in different situations. Similarly, individuals who have been victimised on multiple occasions will have built schemas that might help them detect and respond to these situations. Scripts developed from the perspectives of multiple protagonists (e.g., offender, victim, guardian, etc.) can offer greater insight into the way interpersonal crime occurs and how it can be prevented (Leclerc et al., 2013).

*Script type*: Borrion (2013b) identified three types of offender scripts: *performed* scripts when the offender has committed (or attempted to commit) the crime, *planned* scripts when the offender is considering (how) to commit the crime, and *potential* scripts when they refer to hypothetical crime scenarios. A similar typology can be applied to guardian scripts and victim scripts.

### Script quality

To produce useful results, crime script analysis should preferably start with high-quality scripts. Indeed, no crime analyst would recommend working with scripts containing information that is inaccurate (e.g., actions are incorrectly described or ordered), incomplete (e.g., important actions that are not mentioned in the scripts), irrelevant (e.g., information not useful to the intended application of the script), imprecise (e.g., vague location/context) or ambiguous (e.g., words with multiple or uncertain meanings). For the development of crime reduction measures, for example, reliance on poor-quality scripts could lead to the deployment of ineffective or even counter-effective measures. This is why the issue discussed in the introduction, concerning CSA methods, is fundamental. For an overview of quality criteria see Borrion (2013b).

Quality assurance is relevant not only to individual scripts, but also to the relationships between scripts at different levels of temporal resolution, for instance between meta-scripts and tracks. Although less discussed, congruence between scripts is a topic which must be understood to ensure that scripts can be easily compared and/or integrated together. Indeed, much of the challenge that Borrion et al. (2017) faced in their comparative analysis of 21 scripts stemmed from the fact that the scripts were produced intuitively, without shared decomposition or aggregation rules.

## Theoretical and methodological approach

Production of crime scripts can be seen as a process of deconstruction; not any kind of deconstruction, though, but specifically that which enables preservation or reconstruction of the pattern of behaviour as a meaningful whole which can be related to behavioural processes such as purpose, habit, etc., and their situational contexts (Ekblom & Gill, 2016). In this section, we present the theoretical foundation and methodological approach adopted to examine this problem and outline eight principles that can support the development of more robust crime scripts.

### Modelling framework

#### Procedural units: stages and steps

Schank (1975) describes scripts as structures made of ‘slots and requirements on what can fill those slots’ (p. 117). Cornish (1994a) explains that ‘scripts can be divided up into *scenes*-units of action, or plans required to achieve major sub-goals (Galambos, 1986)—with particular spatiotemporal locations’ (p. 33). To support the decomposition process, he recommends using the universal script, a template comprising nine *scenes* (generalised stages) proposed by Leddo and Abelson (1986, p. 118) and inspired by Schank and Abelson’s (1977) work on knowledge structure in memory: preparation, entry, precondition, instrumental precondition, instrumental initiation, instrumental actualisation, doing, post condition and exit.

Madarie et al. (2024) explain that a range of terms are commonly used to describe the procedural components of crime commission (e.g., act, action, activity, phase, scene, stage and step). We note, for instance, that Cornish and Clarke (2006) did not always adopt the term ‘scene’ in their scripts, preferring to use the more general word ‘stage’ instead. Because our goal is to enhance both the decomposition and aggregation (or abstraction) of these procedural devices, we find it helpful to distinguish between procedural units before and after decomposition. In the following, we refer to these as *stages* and *steps*. A *stage* represents a broader procedural unit prior to decomposition, while a *step* denotes a more fine-grained unit that emerges afterward.

These terms can be applied across different levels of abstraction (metascript, protoscript, script, and track) and temporal resolution (script, act, scene, action). As an example, if an act is described as a stage, a scene of the act would be a step. Furthermore, since steps themselves can be further broken down, a procedural unit (e.g., ‘prepare a kettle of hot water’) can be treated as a *step* in relation to the longer stage it belongs to (e.g., ‘make a cup of tea’), but also as a *stage* in relation to its constituting steps (e.g., ‘turn on the tap’). One of the main advantages of defining relative terms (*stages and steps)* is they are applicable at any level of resolution and would still work even if new levels are added to the aforementioned classifications.

*States and activities*.

The main contribution of this paper is inspired by systems theory (Dekkers, 2017). The terminology is commonly used in fields such as socio-ecological sciences, engineering sciences (e.g., control theory) and computer science (e.g., agent-based modelling). As explained by Borrion (2013a), crime scripting is also closely related to business process modelling, a field that has considerably advanced modelling notation and devices for industrial applications. For this reason, we also drew on Business Process Model and Notation (BPMN), a standards-based approach to graphical representation for business processes (Object Management Group, 2013). Through this lens, the procedural components of crime scripts can be defined as follows:

- A *state* represents the characteristics of a system or ecosystem at a point in time (Dekkers, 2017). This concept is found in both social and behavioural sciences (e.g., variables in codebooks) and computer science (e.g., attributes in object-oriented programming). It can be applied to any relevant element including, but not limited to actors, props and places: e.g., Offender’s location: outside dwelling).
- An *activity* describes a series of decisions and/or (inter)actions over a period of time. It can be used to explain what happened between two points in time, such as a change of state (e.g., Activity: Offender enters dwelling) or no change other than the passage of time (e.g., Activity: Offender waits outside dwelling for 30 min). The term activity is used in BPMN to refer to ‘work that is performed within a business process’ (Object Management Group, 2013).

Together, states and activities, are essential to the comprehension of the procedural aspects of crime. States can be used to describe situations and offender goals. Activities help us communicate more concisely how and why the states of one or more systems change over specific time periods. They represent clusters of related actions that the cast performs during the crime. Borrion (2013b) refers to both the *states* of the entities involved in a crime event and the *activities* performed by these entities, noting that dependencies between them should be clearly visible (transparency criterion).

### Methodological approach

The development of the decomposition and aggregation principles presented in this paper began with three of the authors compiling a set of observations about the quality of crime scripts. Based on these initial observations, the general approach was defined. Considering scripts as ‘sequences of steps’, ideation was performed in three main directions: *definitional* (what is a step? ), *horizontal*, concerned with the temporal characteristics of the steps within a sequence (start time and duration), the relationships between the steps in a sequence (order, contiguity, overlap); and *vertical*, concerned with the relationships between sequences representing the same crime event at different levels of granularity.

**Fig. 1:**
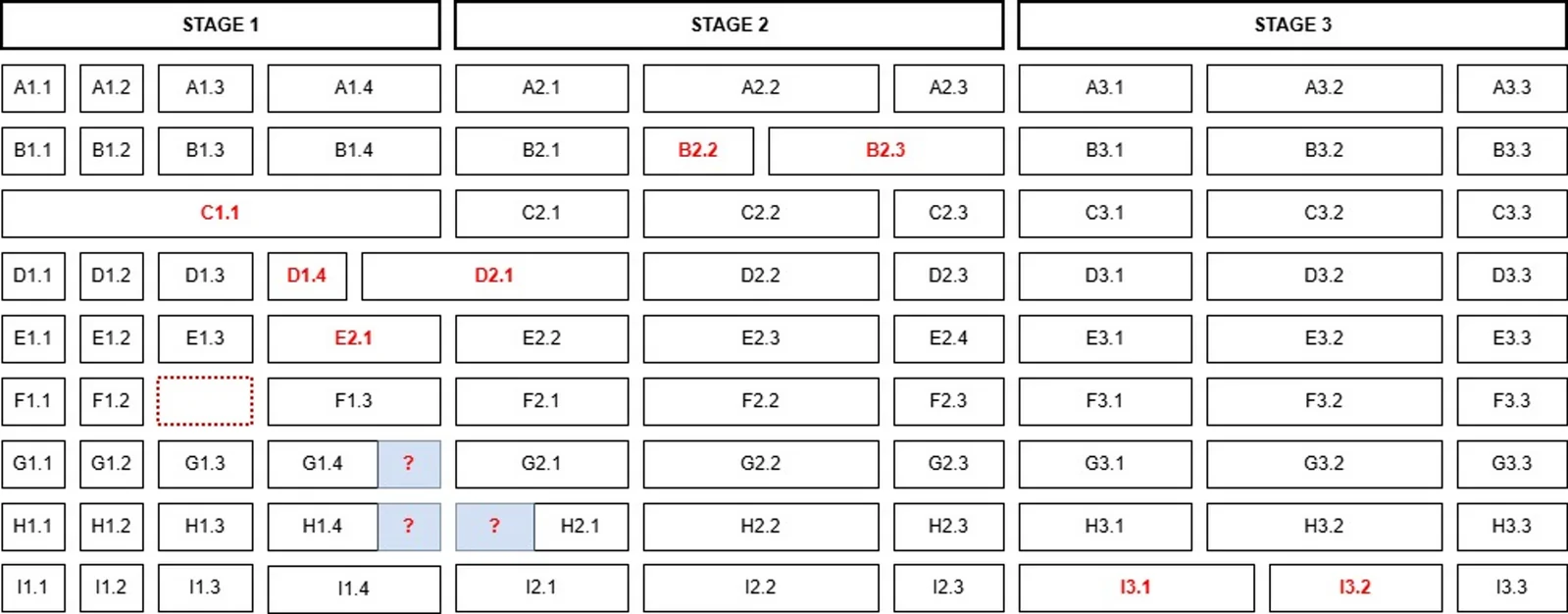
A three-stage script with two almost identical stages, above nine corresponding track variants (A-I). Only Track A adheres to all eight principles

Observations deemed beyond the scope of this paper were removed, and additional ones that could support good practice were incorporated. The resulting set was generated both *inductively*, drawing on several decades of collective experience in creating and reviewing crime scripts, teaching CSA, and using other modelling techniques, as well as *deductively* by examining alternative visual arrangements of procedural steps (Fig. 1). These observations were then discussed and refined into a series of principles explicitly linked to the quality criteria proposed by Borrion (2013b). After this transformation, the principles were reviewed by three additional authors and iteratively revised until consensus was achieved. Comments made by the reviewers of this paper helped refine the principles. The remaining authors subsequently examined the finalised principles and contributed to their validation through the case study.

## Decomposition and aggregation principles

In Table 2, we propose eight principles to help segment stages into steps, or conversely aggregating several steps into stages. We describe them in greater depth below, together with a logical overview of their basis derived from a depiction of shortcomings in a highly abstract script.

**Table 2:** Decomposition and aggregation principles in crime script analysis

Constitution	Each stage and step should represent a transition between two situations
Chronology	Stages and steps should be arranged chronologically
Composition	Each stage should be more abstract and/or last longer than the steps it contains. Each step should be more specific and/or shorter in duration than the stage containing it
Cardinality	Each stage can be associated with more than one step. Each step should be assigned to one—and only one—stage
Categorisation	Steps must be grouped together under the correct stage
Completeness	Scripts should have no missing stages or steps
Clarity	Stages and steps should be clearly described so the reader understands the situations, both before and after
Consistency	Stages that are conceptually similar should be created or decomposed using a similar rule or model

### The logical basis behind the eight principles

The eight principles are illustrated by the track structures in Fig. 1. The latter was generated by conceptualising a script as a sequence of stages and playing with different degrees of freedom: number, position, duration and nature/complexity of the constituting steps. An assumption is made here that the crime script represents a simple sequence of activities.

The first row represents a hypothetical script with three stages; its corresponding track of subsidiary steps (Track A) is immediately below. This is taken as an ideal representation of the script, one which adheres to all eight principles. Since the focus is on sequence structure, the representations are purposely kept very abstract. The position and length of the rectangles in the figure respectively reflect the relative start time, and the duration of the stages and steps.

Track A decomposes each stage into three or four consecutive steps with similar levels of complexity. This track shows that there is no expectation that all stages be decomposed into the exact same number of steps, or that all steps are equally long or equally complex. The next scripts (B-I) show other attempts to represent the *same* event in different ways; they are track variants of the three-stage script, each one illustrating non-adherence to a different principle. To give the simplest illustration of the elements that enable us to discuss the consistency issue, we shall consider that Stages A2 and A3 are almost identical. An example of this might be ‘assault a first target’ and ‘assault a second target’.

### Principles

#### Constitution: each stage and step should represent a transition between two situations

Scripts are portrayed as structures made of slots (Schank, 1975, p. 117) that represent sequences of steps (Cornish 1994b). To model and analyse crime events effectively, researchers need a clear understanding of what the building blocks of these structures represent.

According to the **constitution** principle, each stage and each step should represent a transition from one situation to another, usually caused by an activity. Not all changes are caused by the activity, and not all changes are visible. Internal states, such as what an individual knows about a place, their criminal intent, motive and level of motivation are often not directly observable. Nevertheless, mental processes such as internal assessments and cognitive evaluations should be treated as activities since they can cause states to change. Steps in which no significant change occurs, other than the passage of time, can be described using action verbs such as wait, stand, or stay.

**Fig. 2:**
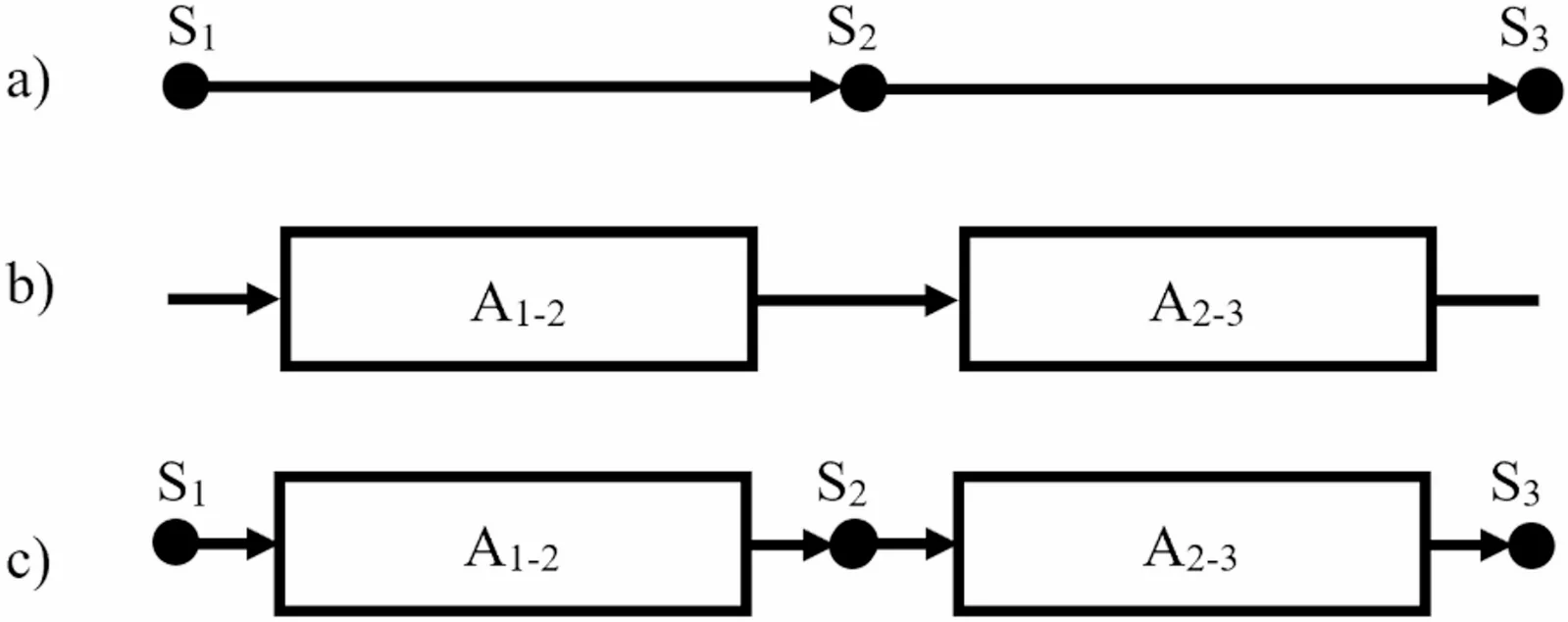
Three possible models for the elaboration of crime scripts:** a** situations only,** b** activities only; and** c** situations and activities (adapted from Borrion & Dehghanniri, 2023)

To build crime scripts, researchers may find the models on Fig. 2 useful. The first two (a, b) are based on the last one (c): [Situation] → (activity) → [Situation]. As explained before, situations can be represented by describing relevant states of the cast, props, place, etc. For example: [Offender location: outside dwelling] → *offender enters* → [Offender location: inside dwelling]. To our knowledge, the first crime script explicitly generated using a state-transition model was an armed robbery script presented at the 2013 Environmental Criminology and Crime Analysis (Borrion 2013a).

#### Chronology: each stage and step should follow chronologically from the one before it

Because crime scripts are ‘procedural sequences’ (i.e., ordered lists of steps), temporal dependency between steps is a central feature of CSA. Chronology underscores that situational crime prevention aims to intervene before the principal act occurs, focusing on preventing or disrupting the causal actions that precede it.

Under the **chronology** principle, stages and steps—along with their associated activities—should be presented in the sequence in which they are believed to occur or have occurred. Accurately capturing this order can be challenging, especially when parts of the event repeat or when the script synthesises several similar incidents. Nevertheless, it should still be possible to specify the ordering between consecutive activities, even when the corresponding script graph contains cycles.

Although Tracks A and B include the same steps, B is less accurate than A because it violates Principle 2. In the crime commission process, Step A2.2 (long step) precedes A2.3 (short step); however, this chronological order is not maintained in Track B. Instead, the two steps are reversed, with B2.2 (which represents the same action as A2.3) incorrectly appearing before B2.3 (which represents A2.2) on Fig. 1.

#### Composition: each stage should be more abstract and/or last longer than the steps it contains. Each step should be more specific and/or shorter in duration than the stage containing it

Both the level of abstraction and duration of procedural units are essential characteristics of crime scripts. Abstraction/generalisation and aggregation enable analysts to make sense of the sequences of actions by situating individual steps within the broader phases they collectively form, thereby revealing how discrete behaviours cohere into a meaningful procedural structure (Ekblom & Gill, 2016). Conversely, refinement/specialisation and decomposition confer the script with the level of specificity needed by analysts to identify the choice-structuring factors that make crime possible or likely (Cornish 1994b).

Following the **composition** principle, each stage should be more abstract and/or last longer than the steps it contains. Each step should be more specific and/or shorter in duration than the stage containing it.

Step C1.1 illustrates a failure to meet the composition principle. It is assumed that Stage 1 represents a complex behaviour, and therefore it should be divided into a set of more specific, simpler or shorter steps to match the other steps in Track C. While varying levels of specificity or duration within a script is not inaccurate, it suggests that little attention might have been paid to some stages, and additional data analysis may be needed to support the identification of effective interventions. Over-coarse steps can straddle distinct causal mechanisms, preventing the attribution of countermeasures to the correct transition. This ‘mechanism conflation’ weakens any prevention logic derived from the script.

#### Cardinality: each stage can be associated with more than one step. Each step should be assigned to one—and only one—stage

The cardinality principle helps ensure that when a stage is broken down into its constituent steps, those steps—when recombined—reconstruct the original stage without adding or losing meaning. Assigning each step to a single stage also has a practical advantage. If different analysts were responsible for different parts of the script, any mismatch between steps and stages across levels of abstraction could lead to duplicated effort (if both analysed the same step) or overlooked content (if each assumed the other was covering it). Although careful task allocation can reduce this risk, it is preferable to avoid it by synchronising steps and stages in the first place.

Each stage can be associated with more than one step. Each step should be assigned to one—and only one—stage. Under the **cardinality** principle, a step must not be linked to multiple stages. If the same action occurs in different stages, it should be represented as distinct steps within each stage.

Track D illustrates a violation of the compartmentalisation principle. Here, D1.4 and D2.1 do not respect the delineation of the high-level script. Because D1.4 ends earlier than A1.4, D2.1 stretches across Stages 1 and 2, making exclusive mapping against any of them impossible.

#### Categorisation: steps must be grouped together under the correct stage

By considering what happens immediately before and after each stage, each step can be placed within the stage that best represents its role in the sequence. This principle reflects the importance given to function and timing in the templates proposed by Cornish (1994b); Tompson and Chainey (2011).

Appropriate **categorisation** implies that steps must be grouped together under the stage that most accurately reflects their timing and influence on the unfolding situation.

Track E illustrates a failure to meet the classification principle. Although it shares the exact same steps as Track A, we can note that the fourth step (E2.1 = A1.4) is incorrectly associated with the second stage. This error can be observed when an action is placed in the wrong row in a tabular script. It may happen for different reasons, when the stages are not well understood (note: this is one of the criticisms of the universal script), or when a track and the corresponding coarser script are generated by different researchers who do not coordinate effectively.

#### Completeness: scripts should have no missing stages or steps

Cornish (1994b) noted that completeness was an important aspect to consider when developing script (see also Borrion 2013b). Providing a script as complete as reasonably possible is important because missing information can make scripts difficult to understand and less useful. For instance, if a step is not included in the script, it is probably less likely that the corresponding pinch points will be identified.

To encourage greater **completeness**, scripts should have no missing stages or steps. When a stage (step) is known to occur but its specific activities are unknown, and its existence is relevant to the purpose of the script, the user should still be informed that the stage (step) is present.

A violation of the completeness principle is illustrated in Fig. 1: Step A1.3 is missing in Track F. According to Cornish (1994b), the identification of missing steps is one of the benefits of using a procedural template based on functional scenes such as the universal script. However, the missing step would potentially remain unnoticed if it was not possible to compare Track F with the ideal track (A).

#### Clarity: stages and steps should be clearly described so the reader understands the situations, both before and after

As explained above, an activity is a concise description (how) and explanation (why) about the state of one or more systems during a given period. It captures what is happening, to whom or what, and how the situation evolves. Because activities typically describe *change*, they need to communicate more than just the action itself. Movement verbs illustrate this well. An action verb like ‘walk’ tells you that someone is moving, but it does not tell you where their trajectory. To ensure that the boundary between steps is clear, the start and end states (e.g., locations) should be specified at least.

Stages and steps should be **clearly** described so the reader understands the situations, both before and after.

In Fig. 1, Track G illustrates a failure to meet the clarity principle. Here, the issue is not so much the alignment between steps and stages, but rather the fact that the situation immediately after G1.4 is not clear. As a result, different researchers may interpret this step differently, meaning that some would be working with an inaccurate script, one that either fails to describe certain actions or that describes actions that did not happen. In some cases, this can be resolved by aligning the (unknown) state at the end of G1.4 with the state at the start of G2.1, when the latter is known.

Track H is another example associated with this principle, this time concerning two contiguous steps. Here, the ambiguity cannot be resolved because both the situation at the end of H1.4 and that at the start of H2.1 are unknown. So, while it is possible to align the timing of these two states to avoid information gaps, it is unclear where exactly the boundary between these two steps lies.

#### Consistency: stages that are conceptually similar should be created or decomposed using a similar rule or model

The last of the eight Cs, consistency, might be seen by some as the cherry on the cake. We argue, however, that following the same rules for decomposing every stage into steps can make scripts more efficient to produce. The adoption of commonly accepted rules can also make scripts less ambiguous.

For **consistency**, stages that are conceptually similar should be created or decomposed using the same (or a similar) rule or model.

Track I illustrates a violation of the consistency principle. As mentioned above, it is assumed that Stages 2 and 3 are almost identical. However, their decomposition yielded very different sequences of steps in Track I. This suggests that stage decomposition was based on different rules or models. If the rule/model underpinning the decomposition of one stage had been applied to both stages, Steps I3.1 and I.3.2 would have resembled I2.1 and I.2.2, respectively, and any improvement made to the shared rule would then benefit all four steps at once.

## Discussion

### Advantages and disadvantages of using templates to create scripts

The principles presented in this paper emerged from a careful review of the CSA literature, including all script-related articles published in this journal to date (see supplementary file), and a return to the foundational sources behind today’s most widely used templates (Cornish 1994a, b; Galambos 1986; Schank 1980; Schank and Abelson 1977). We also examined some of Cornish’s later work and noted that *scenes* were replaced by more specific *stages* (e.g. target selection) (Cornish & Clarke, 2006, 2017). The templates proposed by Tompson and Chainey (2011) and the more recent version by Chainey and Alonso Berbotto (2021) were also important sources for this research.

What this review has revealed is that several features of these templates, often treated as foundational, are in fact pragmatic design choices that have not been theoretically justified. Our aim in this section is not to dismiss them—each template has demonstrably advanced the field—but to argue that the eight principles offer a meta-framework for evaluating them and for supporting more flexible scripting methods that retain the strengths of templates while shedding their arbitrary constraints. Templates clearly have value. They introduce a degree of consistency across scripts, lower the training and cognitive burden for new analysts, and enable cross-study comparison. A problem with existing templates is the conflation of template-specific design choices with the theoretical foundations of CSA itself. Three such conflations are worth examining.

The ‘scene as setting’ reinterpretation. Tompson and Chainey (2011) define a scene as ‘the setting within which the act takes place’ (p.188) (see also Chainey & Alonso Berbotto, 2021). Although the word *scene* (from Greek *skēnē*) originally referred to a physical setting (e.g., *crime scene*), this is not the sense in which Schank and Abelson (1977, p. 42) use the term when they describe *ordering* as ‘one of the scenes’ of their restaurant script. As Cornish (1994a) explains, scenes are ‘units of action, or plans required to achieve major sub-goals (Galambos, 1986)—with particular spatiotemporal locations’ (p. 33). This usage aligns more closely with the dramaturgical sense of the word: ‘part of a play or film in which the action stays in one place for a continuous period of time, or in which the same characters are involved. An act may consist of several scenes’ (Cambridge Dictionary). Tying scenes to physical settings narrows the concept and undermines the principle of containment: the entry and exit scenes of the universal script lose their meaning at sub-scene levels of decomposition, because the actor is typically already in the setting after the entry scene, and they do not need to leave the setting until they reach the exit scene. Scenes, as currently defined in the universal script, are the procedural units resulting from the decomposition of acts, and acts have specific spatial-temporal properties. As such they were probably not designed to be used as part of a recursive decomposition method that could be applied at any level, a problem the relative stage/step terminology proposed in Sect.  3 is specifically designed to avoid. This theoretical point has direct, practical implication. Evidence of this can be found in this journal, with Lavorgna’s (2014, p. 4) assertion that ‘there is no fixed correspondence between the sequence of traditional stages in wildlife trafficking and the sequence of functions’ in the universal script.

Another more substantial reason to consider their method distinct concerns the collapse from temporal-functional to temporal-only categories. Chainey and Alonso Berbotto (2021) divide scenes into ‘four classifications: preparation; pre-activity; activity; and post-activity’ (p. 277), organised by *when* actions occur rather than their functional role. By reducing the number of stages surrounding the main activity from eight to three (before, immediately before, and after), and by replacing generalised scenes with scene classifications that are no longer defined by their functional role in the crime commission process, the revised method becomes easier to grasp. However, as Ekblom and Gill (2016) also argue, such simplification obscures (if not compromises) some of the conceptual structure of Cornish’s (1994b) original method. When activities are categorised by chronology only, the analyst loses access to the functional cues provided by the scenes of the universal script to identify and organise the relevant information, probably increasing the risk of information omission.

Although Cornish’s (1994a) original script template may appear more sophisticated than the simpler versions that followed, we should not conclude that complexity and reduced accessibility for practitioners are its only limitations.

A non-trivial issue concerns the fact that decisions that are treated as theoretical foundations hold in fact pragmatic origins. Both the universal script and its derivatives were shaped by project-specific constraints. Leddo and Abelson (1986) needed 10 scripts of equal length for an experiment (grocery store, painting a room, cashing a check, serving wine, building a campfire, buying a car, pitching a tent, seeing a museum exhibit, going to a ball game, riding a roller coaster); finding Schank’s original seven-stage model too short for their purposes, they split the instrumental plan into three scenes (precondition, initiation, actualisation). Tompson and Chainey (2011) later condensed the resulting nine generalised scenes into four because ‘in comparison to other types of crime, information on illegal waste activity is still scarce’ (p. 187). Both decisions were defensible in their own contexts. Neither was probably intended as a universal foundation. Cornish (1994a) himself acknowledged that ‘adapting the universal script to offending is not without its problems. It is, for example, sometimes difficult to distinguish between preconditions and instrumental preconditions.’

A related issue with existing templates concerns the number of hierarchical levels. The literature distinguishes four levels of abstraction (metascript, protoscript, script, and track), not counting the universal script, and four of decomposition (script, act, scene, action); but neither count is scientifically grounded. As Leclerc et al. (2011) explain, the metascript level comprises ‘all crimes from the same classification (e.g., sexual offending and burglary)’, and the protoscript distinguishes ‘different subgroups of an offense’. At the script level, ‘the offense is then subdivided into categories according to a specific dimension relevant for situational prevention (e.g., victim, situation, and modus operandi)’ (p. 212). But why four? To our knowledge, the hierarchy of categories that underpin these levels is entirely arbitrary, and it can be argued that, regardless of how crimes are classified, intermediate categories can always be inserted. A template-based method that fixes the number of levels of abstraction and decomposition in advance is difficult to justify conceptually and practically.

### Going beyond templates

Our observations suggest that what the field needs is not another template but a more flexible method that can help researchers identify deconstruct crime events, one that retains the chronological and functional cues of the universal script, but can be applied at any level of abstraction and granularity. Within this frame, the eight principles offer a way to guide the emergence of new scripting methods.

Two approaches can be adopted to drive improvement in this area. The first involves evaluating the design of existing or new modelling methods against the different principles. This can be done both when the method is being developed (e.g., during the iterative cycles of design), and once the scripting method is finalised as independent verification. Taking the categorisation principle as an example, a new method could be evaluated by checking if it supports the corresponding rule. Support can take the form of direct explicit guidance (e.g., if it includes a statement such as ‘steps must be grouped together under the correct stage’) or be a property of the design (e.g., if, when researchers follow the method, steps can only be grouped under the correct stage).

The second approach involves evaluating the method’s outputs against each principle. Continuing with the same categorisation example, support for the method may be found in the quality of the outputs (i.e., scripts) generated using the method. However, finding that scripts do not possess the expected properties does not necessarily imply that the method is intrinsically flawed; it could simply mean that the researchers did not apply it properly. The opposite is true too: finding that scripts do possess the expected properties does not necessarily imply that the method is valid. In other words, output-based assessment does not necessarily provide useful evidence to evaluate the method, separately from all other possible factors.

The primary purpose of these two approaches is to assess the merits of scripting methods scientifically and, if needed, improve them or develop new ones that can better satisfy the needs of crime analysts. By adopting the eight principles, a developer can identify and mitigate inherent procedural drawbacks in an existing method, systematically analyse likely performance issues in practice, and draft specifications for next-generation methods to fill existing gaps in a top-down and practical manner.

### Limitations

When using the above eight principles to develop or review scripts (or scripting methods), the reader should be mindful that they were derived inductively, from the authors’ collective experience in creating, reviewing and teaching CSA, and deductively, from systematic analysis of alternative track structures (Fig. 1). Despite our best efforts to ground them theoretically and to illustrate them with abstract counterexamples, several limitations should be acknowledged:

First, the principles were motivated by noticing oddities and anomalies in existing template-based methods. These principles are purposely designed to improve the structuring quality of scripts as models, and support the development of new CSA methods. Because these principles are designed to produce idealised representations of the crime commission process, they may not be applicable to studies that aim to capture raw representations of crime commission, including human errors. This issue is not new, and we found it mentioned by Cornish (1994a): ‘When reconstructing the crime-commission sequence, do we use techniques which elicit scripts, or techniques which may generate or construct them?’ (p.39).

Second, it remains unclear when in the scripting process these principles are best introduced. Applied early in the CSA scripting process, at data collection, they may help experienced researchers produce higher-quality scripts more efficiently by foregrounding key requirements. However, they may divert attention from the primary task of gathering rich, relevant information about the crime. Tentatively, we suggest that the principles are most useful to extract information from the data and assemble it into a coherent procedural model. Furthermore, these principles should be applicable to operationalise some of the evaluation criteria proposed by Borrion (2013b). Empirical work is needed to confirm or revise this recommendation.

Third, although we are interested in how steps can be grouped together (not just how stages can be decomposed), we do not provide a rule for deciding which steps should be combined into a stage. As Cornish (1994b, p. 159) notes, scripts can be divided into scenes that represent ‘smaller units of action or plans required to achieve major sub-goals’. If each stage corresponds to a major sub-goal, see also Morselli and Roy (2008), then grouping steps together means deciding where those sub-goals begin and end. It also means defining the start and end states of the stage itself.

We do not try to resolve this issue in this paper, because doing so would require a fuller account of how sub-goals are identified and justified within a script. This would involve examining not only the structure of action sequences, but also their informational content. Further research is therefore needed, especially on offender sub-goals and how different choices about start and end states affect our understanding of scripts. The concept of states presented in Sect.  3 should be useful for this purpose.

Fourth, the script we had in mind during the development of the eight principles corresponds to one of the simpler forms commonly discussed in the literature: a linear sequence of straightforward activities carried out by a single offender, exemplified by Cornish’s (1994a) burglary script. It is not clear that all eight principles apply perfectly to more complex scripts. Scripts involving multiple interacting actors, branching decision points, or structural features such as repetitions and feedback loops may require certain principles to be relaxed, reinterpreted, or extended. More research should therefore be conducted to examine the applicability of these principles to more complex crime events.

One promising direction is to represent concurrency through parallel columns corresponding to different modalities of activity: physical action (e.g., walking, breaking in), visual perception (e.g., looking inside for signs of occupancy), acoustic perception (e.g., listening for indications that a dog is present), and communication (e.g., asking the co-offender to come downstairs). The principles could then be applied independently within each column, while procedural coherence is maintained across columns. We believe developing and testing this multi-column extension would be a useful direction for future work.

Fifth, the principles still require systematic empirical assessment. Our development work drew in part on a review of published crime scripts, but a more robust evaluation should draw on a broader and more diverse set of scripts. In parallel, the usefulness of the guidance associated with each principle—as prompts for Artificial Intelligence (Large Language Model) applications—should also be examined. Both lines of inquiry are currently underway.

## Conclusion

We began this work with the premise that better guidance was needed to help researchers move from *storytelling*, which simply describes, in an informal and non-replicable way, what the offender(s) did during one or more crime events, to *scripting* that produces procedural models of crime that best support research and practice. Virtually anyone can do the former, perhaps simply by mentally putting themselves in the offender’s shoes and asking ‘what would *I* do?’. But to generate analytic intelligence products (see Snaphaan, 2025) that are reliable, valid and informative requires consistently following (and evolving) rules and techniques and, ideally, doing this as a collective effort across the crime science community.

Effective CSA, like any method, requires expertise. Researchers should be mindful of the characteristics of scripts and of the choices—conscious or unconscious—they make when decomposing a crime event into a step-by-step account of the crime commission process or aggregating multiple actions together. It is also important to recognise how these choices may influence how other researchers interpret a script. If a script is meant to represent multiple events, omitting a step risks being misinterpreted as evidence that an action did not occur, occurred differently than it did, or could occur in ways it cannot.

With this in mind, we have encouraged researchers to conceptualise scripts as a series of situations and activities (that explain how and why the state of entities change); and to adopt relative terms, such as stages and steps, to facilitate the development of recursive decomposition methods. We have proposed eight principles for event decomposition and aggregation that can support not only the writing of crime scripts but also the reading, interpretation, and review of scripts produced by others. These principles are concerned with the following themes: Constitution, Chronology, Composition, Cardinality, Categorisation, Completeness, Clarity, and Consistency. We recognise that, depending on the nature and purpose of the scripts, some of these principles may be challenging to apply or, in certain cases, less than optimal. Our hope is that researchers will test them in practice, identify their strengths and weaknesses, and refine them further to advance CSA methodology.

With this paper, we wanted to both remind the crime science community of the tremendous contribution that Derek Cornish and Ronald Clarke have made to our field, and draw attention to the continuing need to enhance crime scripting and analysis methods. To cite Cornish (1994a), ‘if the elicited scripts are not merely to record the combined abstractions of interviewer and respondent, more attention may have to be paid to these methodological issues than is often the case at present’ (p. 39). What was true then remains true now.

## Supplementary Information

Below is the link to the electronic supplementary material.

Supplementary Material 1.

## Data Availability

Not applicable.
